# Detection of norovirus in stool samples by RT-PCR in 5 disease centers in Iran

**DOI:** 10.1186/1471-2334-12-S1-O10

**Published:** 2012-05-04

**Authors:** Saadat Adabian, Fatemeh Fallah, Latif Gachkar, Farzaneh Jadali, S Rafiei Tabatabaei, Narges Esmaeilnejad

**Affiliations:** 1 Pediatric Infection Research Center, Shahid Beheshti Univ. M.C, Tehran, Iran; 2 Infectious Disease & Tropical Medicine Research Center, Tehran, Iran

## Background

Noroviruses are a group of viruses that cause gastroenteritis (illness that usually includes diarrhea and/or vomiting) in people. Gastroenteritis means inflammation of the stomach and small and large intestines. Many different viruses can cause gastroenteritis, including rotaviruses; noroviruses; adenoviruses, types 40 and 41; sapoviruses; and astroviruses. Current techniques used for detection of noroviruses in stool samples include multi-step viral RNA extraction and purification followed by reverse transcriptase-polymerase chain reaction (RT-PCR). The aim of this study is the detection of norovirus in stool samples by RT-PCR in 5 disease centers in Iran.

## Methods

In this descriptive study, 2170 stool samples of patients consulting for acute gastroenteritis at a pediatric hospital in 5 cities of Iran were enrolled. The mean age of the study population was 48 months with an age range of 30 days to 4 years. Fecal specimens were collected within 24hrs of admission. The specimens were frozen, sent to the laboratory, and then stored at -80°C until being tested for norovirus.

## Results

RT-PCR was evaluated with 2170 stool samples containing 90 (4.14%) norovirus-positive (0.97% Tehran, 0.64% Tabriz, 0.18% Mashhad, 1.57% Shiraz, 0.78% Bandar Abbas). The RT-PCR was validated with published primers for norovirus (JV12/JV13). In both retrospective and prospective settings, the RT-PCR was equally sensitive and specific in detecting norovirus.

## Conclusion

Acute gastroenteritis can be caused by norovirus. It has to be attending to vaccination against norovirus after rotavirus.

